# Evidence for Frozen-Niche Variation in a Cosmopolitan Parthenogenetic Soil Mite Species (Acari, Oribatida)

**DOI:** 10.1371/journal.pone.0113268

**Published:** 2014-11-19

**Authors:** Helge von Saltzwedel, Mark Maraun, Stefan Scheu, Ina Schaefer

**Affiliations:** Georg-August University, Johann-Friedrich-Blumenbach Institute of Zoology and Anthropology, Dept. Ecology, Göttingen, Germany; Nanjing Agricultural University, China

## Abstract

Parthenogenetic lineages may colonize marginal areas of the range of related sexual species or coexist with sexual species in the same habitat. Frozen-Niche-Variation and General-Purpose-Genotype are two hypotheses suggesting that competition and interclonal selection result in parthenogenetic populations being either genetically diverse or rather homogeneous. The cosmopolitan parthenogenetic oribatid mite *Oppiella nova* has a broad ecological phenotype and is omnipresent in a variety of habitats. Morphological variation in body size is prominent in this species and suggests adaptation to distinct environmental conditions. We investigated genetic variance and body size of five independent forest - grassland ecotones. Forests and grasslands were inhabited by distinct genetic lineages with transitional habitats being colonized by both genetic lineages from forest and grassland. Notably, individuals of grasslands were significantly larger than individuals in forests. These differences indicate the presence of specialized genetic lineages specifically adapted to either forests or grasslands which coexist in transitional habitats. Molecular clock estimates suggest that forest and grassland lineages separated 16-6 million years ago, indicating long-term persistence of these lineages in their respective habitat. Long-term persistence, and morphological and genetic divergence imply that drift and environmental factors result in the evolution of distinct parthenogenetic lineages resembling evolution in sexual species. This suggests that parthenogenetic reproduction is not an evolutionary dead end.

## Introduction

Parthenogenetic lineages often are successful colonizers of new or disturbed habitats. This success suggests that effective establishment of populations may occur without males and genetic exchange. In parthenogenetic species each individual represents a reproductive unit capable of founding a new population [1]–[3]. Thelytoky, the exclusive production of daughters from unfertilized eggs, also increases the number of reproductive individuals in a population and thereby population growth. In addition, genotypes that successfully establish in a new habitat are transmitted unchanged to the next generation whereas sexual reproduction potentially breaks up advantageous gene combinations every generation [4]. However, in the long-term, the lack of males and recombination is assumed to result in the accumulation of deleterious mutations [5], [6] and to limit adaptation to changing environments [7], [8]. Therefore, in the long-term parthenogenetic lineages are assumed to be doomed to extinction due to mutational meltdown and competition with sexual sister-taxa.

Among the several hypotheses explaining the ecological and geographical distribution of parthenogenetic and sexual organisms [9] the Frozen-Niche-Variation (FNV) hypothesis [10]–[13] suggests that widespread parthenogenetic species consist of a number of locally adapted genotypes, each occupying a narrow niche. As parthenogenetic genomes are transmitted in full their genotypes are kept “frozen”. In this model asexual individuals arise continuously from sexual populations resulting in genetically diverse populations. Evidence for such specialized genotypes supporting the FNV hypothesis have been found in fishes, frogs, spider mites, shrimps and water fleas [10], [14]–[19]. On the contrary, spatial and temporal variation of ecological niches may favor the evolution of parthenogenetic genotypes adapted to a wide range of ecological conditions, thereby representing a General-Purpose-Genotype (GPG) [2], [20] with only few parthenogenetic lineages dominating across habitats [21]. In these lineages mutations are the primary source of variation [22], [23] resulting in low genetic diversity within populations contrasting predictions of the FNV hypothesis. Evidence for GPG has been found in fishes, snails, ostracods, oribatid mites and ambrosia beetles [24]–[29], for a detailed list see [30].

The cosmopolitan thelytokous oribatid mite species *Oppiella nova* (Oudemans, 1902) lives in a variety of habitats including the soils of forests, grasslands, agricultural fields and suspended soils in tree canopies. It can reach high densities (>20,000 ind. m^−2^) [31]–[34] and often co-occurs with sexual species of the same genus, such as *O. subpectinata* and *O. falcata* [35]. The existence of sexually reproducing congeneric species suggests that *O. nova* is a parthenogenetic offshoot of the predominantly sexual genus *Oppiella* [36]. However, phylogenetic relationships among *Oppiella* species are unresolved and the sexual sister-taxon of *O. nova* is unknown. The most prominent morphological variation in this species is body size which ranges from 220 to 320 µm [37]. Due to morphological variation between habitats Woas [38] suggested *O. nova* to comprise different subspecies each adapted to a distinct habitat.

We analyzed the genetic and morphological variance of populations of *O. nova* from grassland and forest soils, forming two distinct soil habitats likely associated with distinct niches, to investigate whether the variation is driven by FNV or GPG processes. Grasslands and forests differ markedly in abiotic and biotic factors, including temperature, humidity, wind, soil structure and fungal community composition. Mites were sampled along a gradient from grassland to forest at five locations spaced at least 50 km from each other. The mitochondrial *COI* gene and the D3 region of the nuclear 28S rDNA were sequenced to identify genetic lineages; the D3 region also served as species marker [39], [40]. According to the FNV hypothesis we expected specimens of the same habitat to cluster together irrespective of sampling locations. In contrast, conform to the GPG hypothesis different habitats (and the associated niches) within the same location were expected to cluster together, i.e. to cluster according to distance. Although oribatid mites are generally poor dispersers, *O. nova* is able to migrate short distances and occasionally disperses long distances by wind [41]. To take dispersal into account, we tested for migration of genotypes between locations and between habitats, i.e. forest and grassland, within locations. Further, we investigated whether body size correlated with habitat type, genetic lineages or sampling location. Similar to haplotype distribution, we expected body size to correlate with habitat type according to the FNV hypothesis but to correlate with distance of locations according to the GPG hypothesis.

## Materials and Methods

### Ethics statement

Permission for sampling at Kranichstein was given by the forestry office Darmstadt, permission at Hainich was issued by the state environmental office of Thüringen (§ 72 BbgNatSchG). All other sampling sites were outside Nature Reserve Areas and no permission for soil samples was required. The field study did not involve any endangered or protected species.

### Sampling and study sites

**Figure 1:**
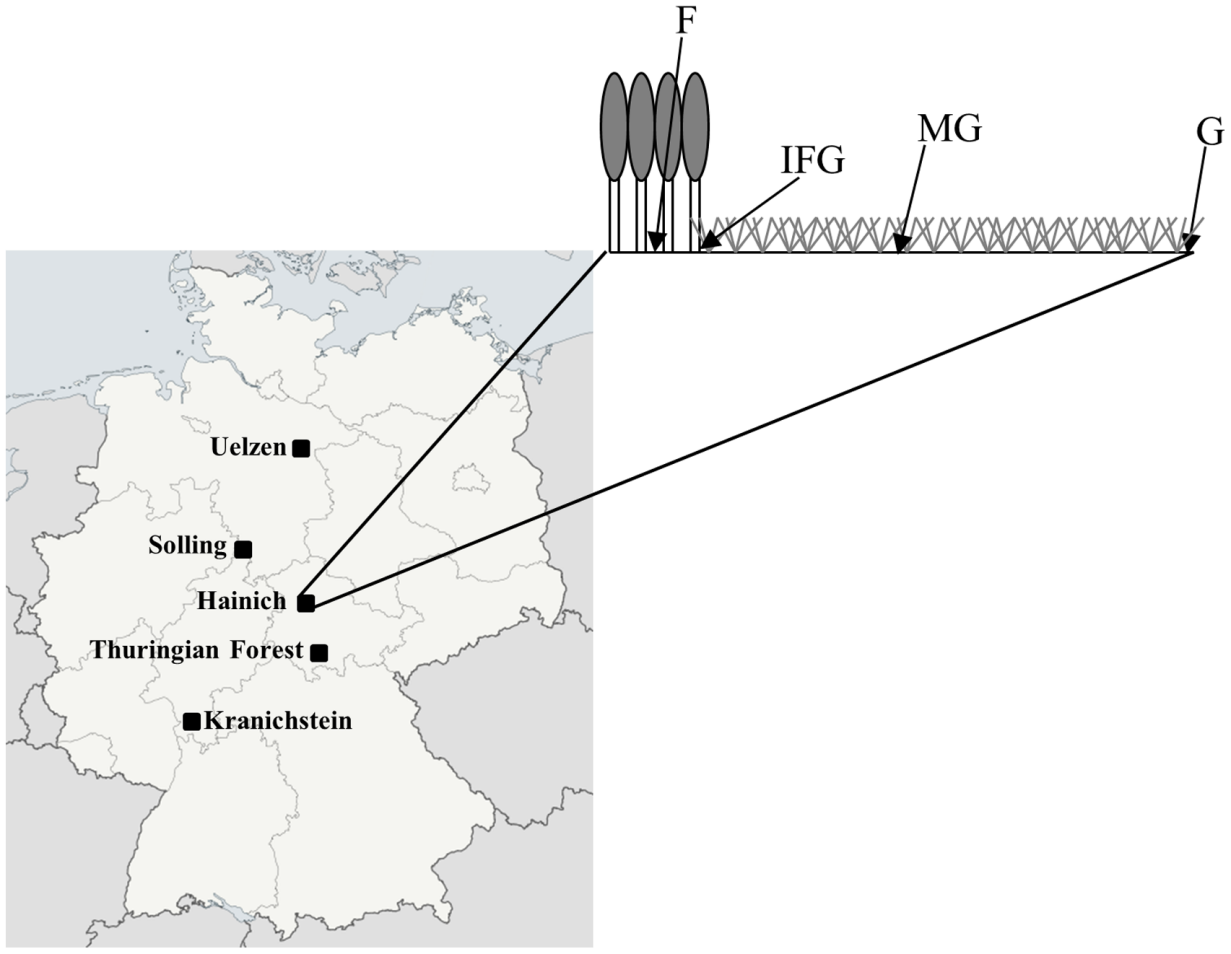
Sampling locations and sampling along a gradient from forest (F) to grassland (G) including transitional habitat types at the intersection between forest and grassland (IFG) and the margin of grassland (MG).

A total of 147 individuals of the oribatid mite species *O. nova* were collected from five locations in Germany: Hainich (HA), Kranichstein (KW), Solling (SO), Thuringian Forest (TW) and Uelzen (UE) (**Table 1** **,** **Figure 1**). We restricted the analysis to the parthenogenetic species because the sexual sister-taxon is unknown. Individuals were sampled from soil and litter of adjacent grassland and forest along a gradient, including the habitat types forest (F) and grassland (G) and two transitional habitats, grassland margin (MG) and intersection of forest and grassland (IFG). MG was located in grassland but close to the forest edge which formed a sharp boundary, IGF samples were taken where tree litter and grassland vegetation mixed (**Figure 1**). The maximum distance between F and G sampling sites was 100 m, MG and IFG sampling sites were 15–20 m apart; sampling locations were 56–350 km apart. From each habitat three samples of 15×15 cm were taken, including litter and the uppermost 5 cm of the soil. Invertebrates were extracted by heat [42] and collected in 75% EtOH. *O. nova* was separated using a dissecting microscope, and morphological identification was confirmed by light microscopy [37].

**Table 1:** Sampling locations, habitat type, number of collected individuals, number of sequences for *COI* and D3 and respective GenBank accession numbers of *Oppiella nova* analyzed in this study.

Location	Habitattype	n indi-viduals	n sequences(*COI*)	GenBank acc. no.	n sequences(D3)	GenBank acc. no.
Kranichstein Forest, nearDarmstadt (KW)	F	17	12	KF293419-26, 35–38,	14	KF293529, 33–41, 51–54
	G	23	19	KF293427-28, 39–55	21	KF293530, 42–44, 55–71
	MG	26	20	KF293415-16, 29–32, 56–69	24	KF293545-48, 72–90
	IFG	4	4	KF293417-18, 33–34	3	KF293532, 49–50
Solling Forest,near Neuhaus (SO)	F	12	7	KF293470-76	8	KF293591-98
	G	1	–	–	1	KF293599
	MG	7	4	KF293477-80	5	KF293600-04
Hainich Forest,near Weberstedt (HA)	F	4	4	KF293402-05	4	KF293514-17
	IFG	12	9	KF293406-14	11	KF293518-28
						
Thuringian Forest, nearIlmenau (TW)	F	3	3	KF293481-83	3	KF293605-07
	G	2	2	KF293484-85	1	KF293608
	IFG	1	–	–	1	KF293609
Uelzen Forest,near Uelzen (UE)	G	4	3	KF293486-88	3	KF293610-12
	MG	7	3	KF293489-91	7	KF293613-19
	IFG	24	20	KF293492-511	20	KF293620-39

Individuals were collected along a gradient from forest (F) to grassland (G), covering the intersection of forest and grassland (IFG) and margin of grassland (MG).

### DNA extraction and PCR

Genomic DNA was extracted from single individuals using the DNeasy Blood and Tissue Kit (Qiagen; Hilden, Germany) following the manufacturer’s protocol for animal tissue. Purified DNA was eluted in 30 µl buffer AE and stored at −20°C until further preparation. All PCR reactions for sequencing were performed in 25 µl volumes containing 12.5 µl HotStarTaq Mastermix (Qiagen; Hilden, Germany) with 1 µl of each primer (10 pM), 1 µl of MgCl_2_ (25 mM) and variable volumes of template DNA (5 µl for D3 and 8 µl for *COI*) and H_2_O (4.5 µl for D3 and 1.5 µl for *COI*). A 709 bp fragment of the *COI* gene was amplified using the primers LCO1490 (forward) 5′-GGT CAA CAA ATC ATA AAG ATA TTG G-3′ and HCO2198 (reverse) 5′-TAA ACT TCA GGG TGA CCA AAA AAT CA-3′ [43]. Amplification consisted of one initial activation step at 95°C for 15 min, followed by 35 amplification cycles of denaturation at 94°C for 30 s, annealing at 40°C for 60 s, elongation at 72°C for 60 s and a final elongation step at 72°C for 10 min. Amplification of the 356 bp fragment of the D3 region of the 28S rDNA was performed using the primers D3A (forward) 5′-GAC CCG TCT TGA AAC ACG GA-3′ and D3B (reverse) 5′-TCG GAA GGA ACC AGC TAC TA-3′ [44]. The PCR protocol for D3 consisted of an initial activation step at 95°C for 15 min, followed by 35 amplification cycles of denaturation at 94°C for 30 s, annealing at 54°C for 45 s, elongation at 72°C for 60 s and a final elongation step at 72°C for 10 min. PCR products were purified with the QIAquick PCR Purification Kit (Qiagen; Hilden, Germany) following the manufacturer’s protocol. Sequencing in both directions (forward and reverse strands) of *COI* fragments was done by Macrogen Inc. (Seoul, South Korea). The D3 fragments were sequenced at G2L (Institute for Microbiology and Genetics, Laboratory for Genomic Analyses, University of Göttingen). All nucleotide sequences are available at GenBank (www.ncbi.nlm.nih.gov/genbank; KF293402 - KF293513 for *COI* and KF293514 - KF293641 for D3).

### Data analysis

Sequences were edited, ambiguous positions were corrected by hand, aided by the respective chromatograms, and nucleotide sequences were translated into amino acid sequences using the invertebrate mitochondrial code implemented in Sequencher 4.9 (Gene Codes Corporation, USA). Consensus sequences were assembled in BioEdit 7.0.1 [45] and aligned with ClustalX v1.81 [46] using multiple alignment parameters: 10.0 (gap opening) and 0.1 (gap extension) for the nucleotide and default settings for the amino acid dataset. In total, three different alignments were generated which included two individuals of *Berniniella hauseri* as outgroup. The D3 alignment included 126 individuals of *O. nova* (**Alignment S1**) and the *COI* alignment 110 individuals (**Alignment S2**). The nucleotide alignments were 356 bp (D3) and 709 bp (*COI*) long; the protein alignment of *COI* had 235 positions.

Phylogenetic trees were calculated with RAxML v8.0.2 [47], MrBayes v3.1.2 [48] and BEAST v1.7.4 [49]. Phylogenetic optimality criterion was maximum likelihood for RAxML, Bayesian inference for MrBayes and BEAST. The best fit model of sequence evolution was estimated with jModeltest 2.1.4 [50], [51], according to the AIC the best model was GTR+I+Γ [52], [53] for both nucleotide alignments. The MCMC chain was run for ten million generations and sampled every 1,000^th^ generation in MrBayes. In BEAST, the MCMC chain ran for 100 million generations and sampled every 10,000^th^ generation, the majority consensus trees were generated with a burnin value of 2,500 (25%). In RAxML 8,000 bootstrap replicates were calculated for statistical node support. A median-joining haplotype network for the nucleotide dataset of *COI* was generated with Network 4.6 (Fluxus Technology, Suffolk, Great Britain).

A strict molecular clock was performed with BEAST v1.7.4, BEAUti v1.7.4 and TreeAnnotator v1.7.4 [49] with a fixed substitution rate of 0.0115 which corresponds to the common invertebrate rate of *COI* of 0.023 substitutions per site per million years [54], [55] for the *COI* nucleotide alignment. The site model was GTR+I+Γ and as tree prior we used “Yule Process” [56] to allow higher rate variation among branches in this parthenogenetic species than coalescent tree priors do. The Yule.birth rate prior had uniform distribution; all priors were estimated by the software. The MCMC chain ran for 20,000,000 generations, every 2,000th generation was sampled and a burnin of 2,500 was applied and convergence of the MCMC was confirmed using Tracer v1.4 [57].

To test for potential migration between forest and grassland and between sampling locations, three models of migration were tested with grassland and forest specific *COI* haplotypes using Bayesian inference in MIGRATE-N 3.2.16 [58]. The three models included (1) panmixis among all locations (50 individuals from forest and grassland) assuming a single population, (2) migration between forest (26 individuals) and grassland (24 individuals), and (3) migration between the five sampling locations. This analyses included 4 individuals from HA, 31 from KW, 7 from SO, 5 from TW and 3 from UE. The models were tested in several independent runs. The following parameters deviated from default settings: 10,000 record steps in chain: heating set to on, static heating; 4 chains sampling at every 10^th^ interval using the temperature scheme suggested with the character #; Theta prior distribution, uniform, 0 (minimum) 1 (maximum) 0.1 (delta); migration prior distribution, uniform, 0 (minimum) 10000 (maximum) 1000 (delta); running multiple replicates set to YES, 4 independent chains; number of long chains to run set to 2. To identify the best-fit model, marginal likelihoods of the three runs were compared by calculating the log Bayes Factor (LBF) and model probability (MP) by substracting the largest log likelihood from every other log likelihood, exponentiating the difference and summing up the results. The exponential elements were divided by this product; results indicate which model is most likely relative to the others [59].

Two independent analyses of molecular variance (AMOVA) were calculated in ARLEQUIN 3.5 [60] to investigate between and within population structure based on p-distances, selecting (1) habitat (forest and grassland) and (2) sampling locations as group. Populations represented by less than three individuals were excluded. According to the FNV hypothesis we expected higher variance within sampling locations (i.e., high variance between habitats and associated niches) than between sampling locations, whereas according to the GPG hypothesis we expected variance within habitats to be similar or lower than between sampling locations. Isolation by distance was tested by Mantel test implemented in ARLEQUIN using 10,000 permutations and straight-line (Euclidian) distances. Haplotype and nucleotide diversity were also calculated in ARLEQUIN. To distinguish between divergent selection and neutral drift the distribution of synonymous and non-synonymous substitutions between locations and between habitat types was compared using the McDonald Kreitman test [61] in DnaSP v5.10.1 [62].

For morphological variation body length and width of 147 individuals were measured from dorsal pictures, taken with AxioCam HRm and processed with the image analyzing software AxioVision 4.8.2 (Zeiss, Göttingen, Germany) by quantifying pixels. Differences between mean values of body length of *O. nova* were analyzed in R 3.1 (R Development Core Team 2014) using the linear mixed effects model (nlme package) [63]. Locations were set as random variable and body-sizes were compared between habitat types (F, G, IFG, MG) and additionally between individuals with forest and grassland specific *COI* genotypes. *Post hoc* multiple comparisons of means were made using Tukey’s honestly significant difference test (multicomp package) [64] with p<0.001 as threshold for significance.

## Results

**Figure 2:**
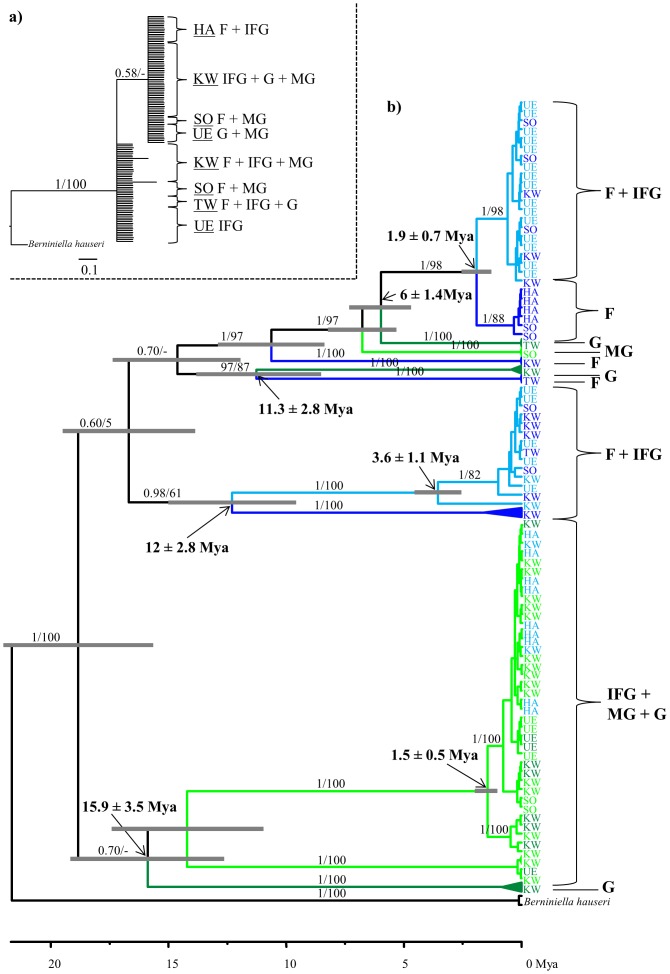
Bayesian phylogenetic trees showing the relatedness among individuals of *Oppiella nova* from different habitats based on (a) nuclear (D3 region of the 28S rDNA, 126 individuals) and (b) mitochondrial markers (*COI*, 110 individuals). Different colors indicate *COI* haplotypes of forest (F, blue), grassland (G, green), intersection of grassland and forest (IFG, light blue), and margin of grassland (MG, light green). Numbers on nodes represent posterior probabilities and bootstrap values, bold numbers are median estimated divergence times ±95% HPD calculated in BEAST using a strict molecular clock and grey bars on nodes indicate 95% HPD intervals. UE (Uelzen), SO (Solling), HA (Hainich), TW (Thuringian Forest) and KW (Kranichstein Forest) refer to the locations of the five forest – grassland gradients studied.

Densities of *O. nova* varied between zero and 30 individuals per sample. To obtain equal numbers of individuals per habitat type, the three samples of each sampling location were pooled for further analysis. Numbers of individuals at the four habitat types were 36, 30, 40 and 41 for F, G, MG and IFG, respectively. Molecular variation of the D3 fragment was low; only nine positions of the 354 bp fragment varied in 126 analyzed individuals (positions 59–61, 114 and 120–124). Accordingly, the phylogenetic tree had no structure and habitats and locations were mixed (**Figure 2a** **, Figure S1–2**). Amino acid sequences of the *COI* fragment were almost identical in the 110 individuals sequenced. Only 16 specimens had one or two variable sites with non-synonymous substitutions (**Table S1**) and the overall genetic distances between protein sequences were low (<0.5%). In each of the phylogenetic trees *O. nova* was monophyletic and separated with high support from the outgroup taxon *B. hauseri*. Trees (MrBayes, BEAST and RAxML) based on the *COI* nucleotide alignment were similar (**Figure S3–4**) and *COI* haplotypes generally clustered according to habitat type, irrespective of sampling locations (**Figure 2b**). Applying a mitochondrial substitution rate of 2.3% per million years, F and G lineages diverged between 6 and 16 mya (**Figure 2b**).

**Figure 3:**
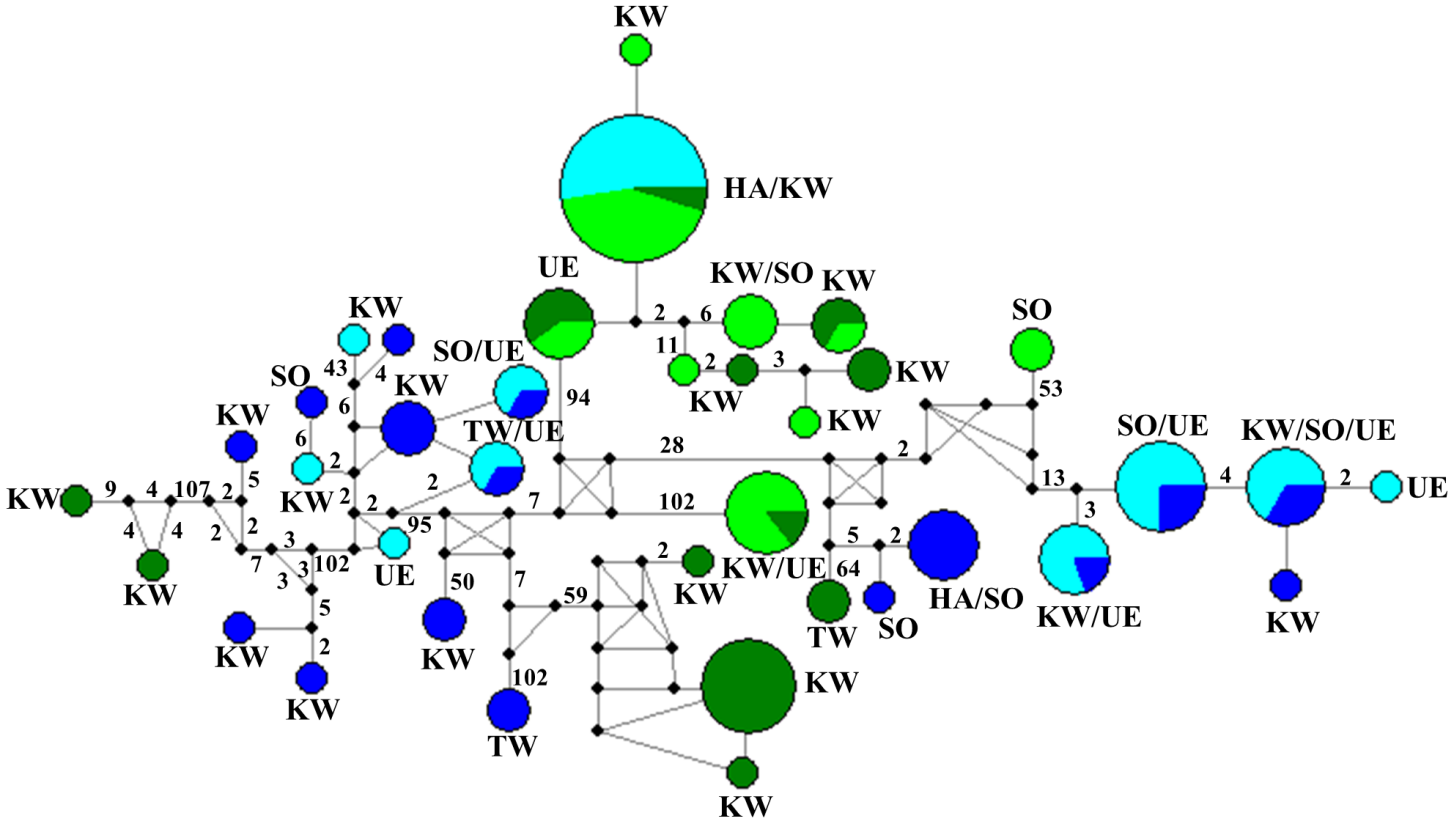
Median Joining Haplotype Network of 110 *COI* sequences of *Oppiella nova* collected from forest (blue), intersection of grassland and forest (light blue), margin of grassland (light green) and grassland (green) from five sampling locations (UE, Uelzen; SO, Solling; HA, Hainich; TW, Thuringian Forest; KW, Kranichstein Forest). The size of circles is proportional to the number of sequences per haplotype. Numbers on lines represent mutation steps separating the haplotypes; no number indicates a single mutation step. Haplotypes from forest and grassland are always separated by many mutation steps, but either haplotypes from forest or from grassland are closely related, even from distant sampling locations.

The *COI* haplotype network also showed a strong habitat related structure (**Figure 3**). Individuals from several sampling locations had identical or closely related haplotypes. However, individuals from forest (F) and grassland (G) had distinct haplotypes, irrespective of sampling locations, i.e., individuals from F and G always clustered separately. Haplotype from IFG either clustered with individuals from G or F, individuals from MG either clustered with individuals from G or IFG (except for two individuals from Solling that formed an isolated clade).

In total, 37 haplotypes were sampled and one haplotype was very common with a total of 21 individuals, 11 from IFG, 9 from MG and one from G. Haplotypes from IFG commonly occurred in more than one habitat type, F and IFG shared five, G and MG shared four common haplotypes, whereas IFG and MG as well as G and IFG shared only one haplotype. In contrast, no haplotypes were shared between F and MG as well as between F and G. Haplotype (Hd) and nucleotide (Π) diversity showed similar patterns (**Table S2**). Within sampling locations Hd of the four habitat types was similar, being between 0.8–0.96. In HA, both Hd and Π were lowest, in SO haplotype diversity was highest (Hd = 0.95) but nucleotide diversity was only intermediate (Π = 0.1). Haplotypes in other locations and in F and G were more different from each other.

As indicated by AMOVA, genetic variance was generally high, being highest within samples (58%) and lower within locations (43%) and lowest within habitat types (35%) (**Table 2**). The negative variance component among locations resulted from low or nearly absent genetic structure. If the expectation of the estimator is zero, AMOVA can generate slightly negative variance components.

**Table 2:** AMOVA of the *COI* gene of *Oppiella nova* on the variance among and within locations and among and within habitat types.

source of variation	d.f.	sum of squares	variancecomponents	percentage ofvariation	fixiation indices	
Among locations	4	874	−0.40 Va	−1	Fct	−0.01
Among habitats	3	1,124	3.20 Va	6	Fct	0.06
Among samples within locations	8	1,609	22 Vb***	43	Fsc	0.43***
Among samples within habitats	9	1,308	19 Vb***	35	Fsc	0.39***
Within samples	97	2,898	30 Vc***	58	Fst	0.42***

Within samples variance was identical for both analyses; asterisks indicate significant differences at p<0.001; d.f. are degrees of freedom.

Among the three models tested with MIGRATE-N, migration between locations (model 3; log marginal likelihood = −5066, LBF = 0, MP = 1) was most likely. Substantially less likely were migrations between F and G (model 2; log marginal likelihood = −5317, LBF = −502, MP = 9.8E-110) and panmixis (model 1; log marginal likelihood = −5259, LBF = −386, MP = −1.5E-84). Isolation by distance was rejected being not significant (r(Y) = −0.24, P(rY) = 0.98). The McDonald Kreitman test was not significant for all comparisons as non-synonymous substitutions were not fixed within habitat types or locations.

**Figure 4:**
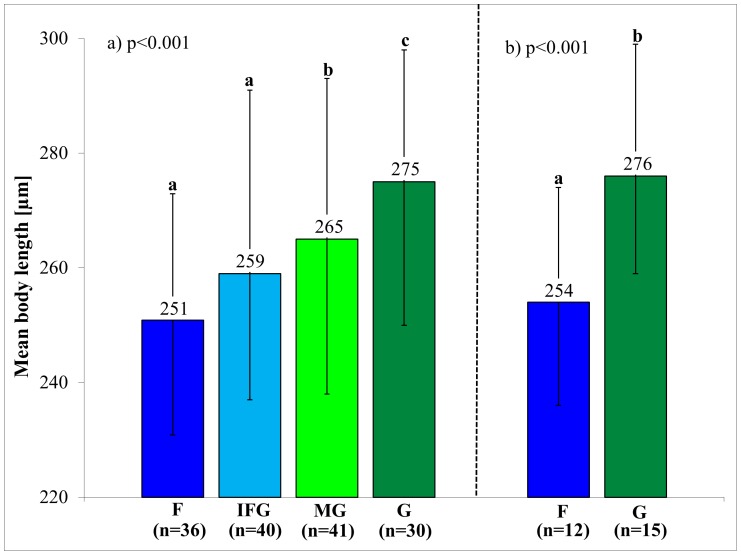
Body length of *Oppiella nova* from forests, grasslands and transitional habitat types between forests and grassland. (a) Differences between all collected individuals (147); forest (F), grassland (G), intersection of forest and grassland (IFG), and margin of grassland (MG). (b) Differences in body size between individuals with forest and grassland specific genotypes. Numbers in brackets refer to the number of individuals included in the analyses; error bars indicate standard deviation. P-values correspond to Tuckey’s HSD test.

Body length of *O. nova* (**Table S3**) in the different habitats ranged from an average of 251 to 275 µm with individuals from G being 24 µm longer than those from F (F_3,139_ = 23.83, p<0.001 for habitat type; **Figure 4**). Accordingly, body length of individuals with forest and grassland specific genotypes differed significantly (F_1,22_ = 22.06, p<0.001). Body size of individuals from IFG and F was similar; MG and IFG were in between that of individuals from F and G.

## Discussion

The results indicate that *O. nova* differs both genetically and morphologically between forest and grassland. In agreement with the FNV hypothesis, haplotypes of forest and grassland were distinct and formed well-supported grassland and forest clades. Although individuals from both habitats were always distinct, some haplotypes also occurred in the transitional habitat types IFG and MG. This suggests niche-related environmental filtering between forest and grassland haplotypes with forest and grassland haplotypes coexisting in transitional habitats. Notably, forest and grassland haplotypes significantly differed in morphology with body size gradually increasing with distance from forest reaching a maximum in grassland specimens.

Considerable molecular variance was found in each of the locations and habitat types, suggesting independent colonization by different lineages rather than by a single locally adapted lineage. High molecular variance within sampling locations suggests that different lineages exist in neighboring habitat types at each sampling location. The results indicate that forest and grassland habitats are associated with certain niches selecting for specific genotypes with both niches being present in transitional habitats, which is consistent with the FNV model. Notably, haplotypes present in more than one habitat type also occurred at different locations. These widespread haplotypes predominantly colonized transitional habitats but haplotype diversity in these habitats was generally lower than in forests and grasslands. Environmental conditions in transitional habitats probably favor more generalist genotypes.

According to ecological niche theory, interspecific competition favors the evolution of species occupying separate niches. Species performance therefore is limited by environmental conditions and genetic adaptation, restricting geographic distribution. Similarly, intraspecific differentiation also can be linked to divergences in environmental conditions or resources. Niche differentiation typically is manifested in morphological differentiation, but may also be cryptic and only recognizable at physiological, genetic or transcriptomic levels [65]–[68]. Difference in body size is a common feature that separates individuals along a single resource dimension [69], whereas genetic differentiation is usually correlated with reproductive or geographic isolation [70]–[72].

In *O. nova* isolation by distance was not significant and differences in body size correlated with separation into forest and grassland, indicating that niche specific size-dimorphism is due to habitat specific adaptations rather than geographical differentiation. Variation in body size likely reflects niche differentiation, which often is induced by resource shifts and differential exposure to predators [73]–[75]. Stable isotope data from oppiid species indicate that *O. nova* lives as predator or scavenger [76], [77] and size dimorphism therefore may reflect adaptation to prey of different body size. However, differences in habitat structure and different predator communities in grasslands and forests may also be responsible for the observed variations in body size. Adult oribatid mites typically are well protected from predation by morphological and chemical defenses [78]–[80]. However, Schneider and Maraun [81] demonstrated that gamasid mites, the most vigorous predators of soil microarthropods, prey heavily on *O. nova.* Gamasid mites preferentially prey on oribatid mite species of a body size of 200–300 µm [81], indicating that larger and smaller species live in size refuges. Large oribatid mites are heavily sclerotized while smaller ones and juveniles typically are weakly sclerotized but colonize pore space inaccessible for predators such as gamasid mites. For *O. nova*, which is small, weakly sclerotized and mobile, top-down control by gamasid mite predators is likely to be important with larger individuals suffering less from predation by gamasid mites than smaller ones. Differences in body size in forest and grassland therefore may reflect body size related differences in predation by gamasid mites. Unfortunately, little is known on the control of oribatid mites by gamasid mite predators in the field and whether this differs between forest and grassland.

Overall, our data indicate ecological differentiation of a parthenogenetic lineage into discrete genetic and morphological entities. The gradual change in haplotype composition and body size between forest and grassland indicates adaptation to specific environmental conditions, i.e. a shift in ecological niches. Further, the results suggest that in addition to haplotypes from both forest and grassland, transitional habitats are colonized by widespread genotypes with lower haplotype diversity than forest and grassland. In contrast to forests and grassland, oribatid mites of transitional habitats may be less affected by predation but rather by abiotic forces due to more variable climatic conditions. Despite the distinctness of forest and grassland lineages and non-synonymous substitutions in the *COI* gene, no indications for divergent selection were found. This may be due to the large population size of *O. nova* as genetic drift and fixation probability of mutations decrease with increasing population size. *Oppiella nova* is among the most abundant oribatid mite species in grasslands and forests and can reach densities of thousands of individuals per square meter [82], [83], [34]. This suggests that extinction rates and bottlenecks are of minor importance explaining why genetic variance of the *COI* fragment within populations is high. Despite separation of shallow clades by long branches in the mitochondrial dataset, which may indicate a cryptic species complex, low D3 variance suggests that *O. nova* may best be treated as single (parthenogenetic) species. High intraspecific *COI* variance is common in arthropods [84], [85], especially in those living in soil [86]–[88], including parthenogenetic oribatid mites [28] and bdelloid rotifers [89].

In contrast to *O. nova*, haplotype diversity in the parthenogenetic oribatid mite *P. peltifer* suggested a general purpose genotype [28]. *Oppiella nova* is a fast reproducing [90] weakly sclerotized r-strategist [91] presumably feeding on living resources and therefore subject to co-evolutionary adaptations [76]. In contrast, *P. peltifer* reproduces slowly and is strongly sclerotized, characters typical for K-strategists. It predominantly feeds on dead organic matter suggesting that co-evolutionary processes between consumer and (dead) food resource are non-existing [91], [77], thereby facilitating more generalist genoptypes.

Our age estimations suggest that lineages of *O. nova* from grassland separated from those of forests during the Middle and Late Miocene (16-6 mya). The substitution rate of parthenogenetic species may differ from the general rate of *COI* established for arthropods. Still, age estimates and high genetic distances between forest and grassland lineages suggest long-term separation and persistence of lineages, contradicting the commonly held view that parthenogenetic lineages are short-lived evolutionary dead ends. Speciation of parthenogenetic lineages has been assumed to be responsible for the formation of large phylogenetic clusters in bdelloid rotifers [92]–[94] and certain groups of oribatid mites [40], [95]. The age of grassland lineages correlated well with the expansion of grasslands in the Miocene [96], [97] indicating long-standing adaptation to this habitat. Present day occurrence of grassland and forest lineages in managed European grasslands and forests, respectively, suggests recurrent establishment of lineages due to environmental filtering, i.e. grassland and forest lineages remained bound to the respective habitats.

Our results suggest that, as in sexual species, environmental filters and biotic interactions contribute to the evolution of parthenogenetic species. High genetic variability presumably is maintained by adaptation of certain genotypes to environmental settings as suggested by the FNV hypothesis. Habitat partitioning and coexistence of parthenogenetic lineages at local scales suggest that speciation may occur sympatrically.

## Supporting Information

Figure S1 **Maximum Likelihood tree showing the relatedness among individuals of** ***Oppiella nova*** **from different habitats based on nuclear marker (D3 region of the 28S rDNA, 126 individuals). UE (Uelzen), SO (Solling), HA (Hainich), TW (Thuringian Forest) and KW (Kranichstein Forest) refer to the locations of the five forest – grassland gradients studied (see** **Figure 1** **).**
(TRE)

Figure S2 **Bayesian phylogenetic tree showing the relatedness among individuals of** ***Oppiella nova*** **from different habitats based on nuclear marker (D3 region of the 28S rDNA, 126 individuals). UE (Uelzen), SO (Solling), HA (Hainich), TW (Thuringian Forest) and KW (Kranichstein Forest) refer to the locations of the five forest – grassland gradients studied.**
(TRE)

Figure S3 **Maximum Likelihood tree showing the relatedness among individuals of** ***Oppiella nova*** **from different habitats based on mitochondrial marker (** ***COI*** **, 110 individuals). UE (Uelzen), SO (Solling), HA (Hainich), TW (Thuringian Forest) and KW (Kranichstein Forest) refer to the locations of the five forest – grassland gradients studied.**
(TRE)

Figure S4 **Bayesian phylogenetic tree showing the relatedness among individuals of** ***Oppiella nova*** **from different habitats based on mitochondrial marker (** ***COI*** **, 110 individuals UE (Uelzen), SO (Solling), HA (Hainich), TW (Thuringian Forest) and KW (Kranichstein Forest) refer to the locations of the five forest – grassland gradients studied.**
(TRE)

Table S1 **Non-synonymous amino acid substitutions among** ***COI*** **sequences of Oppiella nova from F, G, IFG and MG.** Non-synonymous substitutions are highlighted in red and positions in the *COI* fragment are indicated; individuals affected are from Kranichstein Forest (KW), Thuringian Forest (TW) and Uelzen (UE).
(XLSX)

Table S2 **Number of individuals (n Ind), number of haplotypes (n haplo), haplotype (Hd) and nucleotide diversity (π) of habitats and locations of Oppiella nova.**
(XLSX)

Table S3 **Body length [µm] of all individuals of** ***Oppiella nova*** **sampled for this study and are included in** **Fig. 4a** **.**
(XLSX)

Alignment S1 **Alignment of the 28S rDNA (D3 region; 356 bp) including 126 individuals of** ***O. nova*** **.**
(FAS)

Alignment S2 **Alignment of the** ***COI*** **gene (709 bp) including 110 individuals of** ***O. nova*** **.**
(FAS)
